# Molecular Variation and Genomic Function of Citrus Vein Enation Virus

**DOI:** 10.3390/ijms24010412

**Published:** 2022-12-27

**Authors:** Runqiu Dou, Qingqing Huang, Tao Hu, Fengzhe Yu, Hongxia Hu, Yaqin Wang, Xueping Zhou, Yajuan Qian

**Affiliations:** 1 State Key Laboratory of Rice Biology, Institute of Biotechnology, College of Agriculture and Biotechnology, Zhejiang University, Hangzhou 310058, China; 2 State Key Laboratory for Biology of Plant Diseases and Insect Pests, Institute of Plant Protection, Chinese Academy of Agricultural Sciences, Beijing 100193, China

**Keywords:** citrus vein enation virus, P0 protein, phase separation, hypersensitive response, suppressor

## Abstract

In this study, we identified a new citrus vein enation virus (CVEV) isolate (named CVEV-DT1) through sRNA high-throughput sequencing and traditional sequencing. Phylogenetic analysis based on whole genome sequences of all known CVEV isolates revealed that CVEV-DT1 was in an evolutionary branch with other isolates from China. Molecular variation analysis showed that the single nucleotide variability along CVEV full-length sequences was less than 8%, with more transitions (60.55%) than transversions (39.43%), indicating a genetically homogeneous CVEV population. In addition, non-synonymous nucleotide mutations mainly occurred in ORF1 and ORF2. Based on disorder analysis of all encoded ORF by CVEV-DT1, we identified that the CVEV-DT1 coat protein (CP) formed spherical granules, mainly in the cell nucleus and partly throughout the cytoplasm, with liquid properties through subcellular localization and photobleaching assay. Furthermore, we also confirmed that the CVEV P0 protein has weak post-transcriptional RNA-silencing suppressor activity and could elicit a strong hypersensitive response (HR) in tobacco plants. Collectively, to the best of our knowledge, our study was the first to profile the genomic variation in all the reported CVEV isolates and reveal the functions of CVEV-DT1-encoded proteins.

## 1. Introduction

*Enamovirus* and *Polerovirus* are two closely related genera in the family *Solemoviridae*, which were previously classified as being in the family *Luteoviridae* [1]. The main differences between the *Enamovirus* and *Polerovirus* genera involve their nucleotide sequence and genomic organization [2]. In terms of the positive-sense genomic sequences, the single-stranded RNA genome ranges from 5.6 to 6.2 kb. Commonly, poleroviruses and enamoviruses contain similar genomic organization features, encompassing an RNA-dependent RNA polymerase (RdRp) that is a translational fusion of ORF1 (P1) through ORF2 (P2), a coat protein (CP, encoded by ORF3), a P0 protein encoded by ORF0 and a read-through coat protein (CP-RTD, encoded by ORF5). The main distinctive feature of enamoviruses is lack of movement protein in the genome.

Citrus vein enation virus (CVEV) belongs to genus *Enamovirus*, which currently contains five species. The full-length genome sequences of CVEV range from 5979 to 5983 nt in length, containing five open reading frames (ORF0, ORF1, ORF2, ORF3 and ORF5) in an overlapping mode and two untranslated regions. CVEV is the causal agent of citrus vein enation disease, which is graft- and aphid-transmissible [3,4,5]. The citrus vein enation disease was first discovered in sour orange in California, in the United States in 1953, and it was then identified in South Africa [6,7]. The main symptoms associated with citrus vein enation disease were characterized by the formation of small outgrowths in the abaxial surface of leaves and corresponding indentations in the adaxial surface, mainly on the secondary leaf veins, as well as galls on the trunk and branches [8,9]. At present, the disease is widely distributed in citrus-growing areas in Brazil, Spain, Japan, Australia, Brazil, Korea, India, Turkey, Iran and Peru, affecting sensitive citrus species and causing serious losses to the citrus industry [3,10,11,12,13]. Since it was first detected in Huangyan, Zhejiang Province, China in the 1990s, the disease has been reported in several citrus-producing areas in China [14,15].

RNA silencing is the main defense mechanism against plant viral infection. RNA silencing is generally divided into transcription-level gene silencing (TGS) and post-transcriptional gene silencing (PTGS) according to the different targets [16,17]. TGS mainly targets DNA in the nucleus [17]. PTGS occurs at the RNA level to degrade specific mRNA sequences through RNA-induced silencing complexes, which carry Argonaute proteins (AGOs) and small RNA molecules [18]. Multiple P0 proteins derived from different poleroviruses have been shown to act as PTGS suppressors that leads to the degradation of AGO1 by an autophagy-related process [19]. Besides playing an important role in RNA silencing, several P0 proteins from poleroviruses were found to induce the cell death phenotype in inoculated tobacco leaves [20]. A recent study confirmed that the P0 protein encoded by pea enation mosaic virus 1 (PEMV1), which is a member of the genus *Enamovirus*, could inhibit local and systemic PTGS [21]. To our knowledge, this is the first report of an RNA-silencing suppressor related to enamoviruses. However, to date, it is not known whether P0 enamoviruses could serve as an inducer of the cell death phenotype.

Multiple complete nucleotide sequences of CVEV have been successively reported; however, little is known about the genetic variation across the CVEV genome. Additionally, the biological functions of CVEV-encoded proteins are still largely unknown. In this study, we identified a new CVEV isolate (named CVEV-DT1) from a citrus sample collected from Zhejiang Province, China, through high throughput sRNA sequencing and traditional Sanger sequencing. Subsequently we profiled genomic variation in all reported CVEV isolates, containing an analysis of single nucleotide variability and a mutational map. Importantly, detection of sequence disorder in all five ORFs of CVEV-DT1 and subcellular localization strongly indicated that CP could form large granules and might be involved in phase separation. In addition, we further investigated the role of P0 protein encoded by CVEV-DT1 in suppressing PTGS and inducing HR-like cell death in tobacco leaves. Our findings provide insights into the CVEV population variation, as well as facilitating our understanding of the pathogenicity of CVEV.

## 2. Results

### 2.1. Small RNA Deep Sequencing and CVEV-DT1 Identification

**Figure 1:**
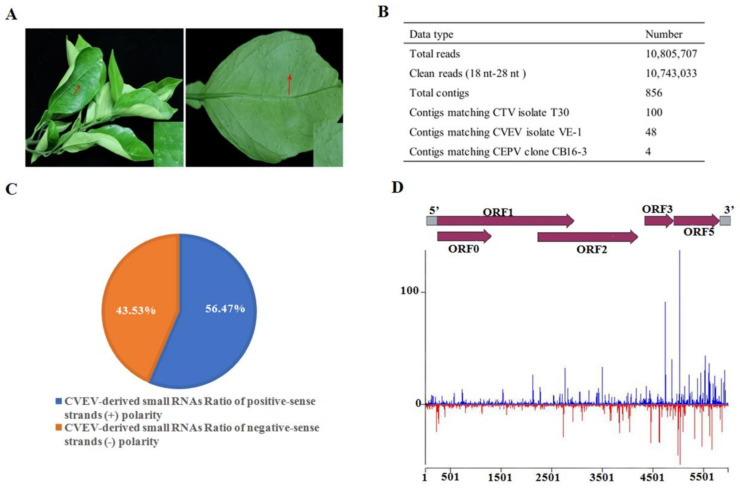
Identification of citrus vein enation virus (CVEV) and viral-derived siRNAs. (**A**) Typical symptoms represented in a leaf associated with vein enation disease. (**B**) Contigs assembly derived from sRNAs sequencing database. (**C**) Polarity distribution of vsiRNAs mapped to positive-sense or negative-sense CVEV genomic sequences. (**D**) Distribution of vsiRNAs along the CVEV-DT1 genome.

One field citrus leaf sample showing vein enation symptoms from Lishui district of Zhejiang Province, China, was analyzed using high-throughput Illumina sequencing (Figure 1A). A total of 10,743,033 clean reads between 18 nt and 28 nt were obtained and subjected to de novo assembly. Subsequently a BLASTn (nucleotide BLAST) search in the National Center Biotechnology Information (NCBI) database confirmed that 152 contigs were mapped to the genomic sequences of citrus tristeza virus (CTV), CVEV and citrus endogenous pararetrovirus (CEPV), respectively (Figure 1B). Only sequences derived from CVEV were further analyzed in the present study.

RT-PCR analysis using virus-specific primers confirmed the existence of CVEV in the DT1 sample (Figure S1). To further obtain the complete sequence of the CVEV-DT1 isolate, six overlapping fragments were amplified by RT-PCR using primer pairs designed based on four contigs mapped to the CVEV genome and the known CVEV-VE1 sequence, and they were sequenced. After these PCR products were assembled into a contiguous sequence, the 5-terminal and 3-terminal sequences were determined through RACE amplification. Finally, the full-length sequence of the CVEV-DT1 isolate was determined to be 5983 nt (GenBank: ON494593). A BLASTn search using the complete sequence of CVEV-DT1 in NCBI revealed that it shared the highest nucleotide similarities (97.9%) with the isolates of CVEV-XZG and CVEV-SM, derived from China (GenBank: KY303624.1 and MN596377.1).

SnapGene analysis predicted five ORFs in the genome of CVEV-DT1, including ORF0 (1065 nt), ORF1 (2709 nt), ORF2 (3971 nt), ORF3 (576 nt) and ORF5 (1485 nt), which is in accordance with the genomic organization predicted by other known enamoviruses. The P0 protein encoded by ORF0 is a 39 kilodaltons (kDa) protein composed of 354 amino acids. ORF1 encodes a 100 kDa polypeptide of S39 serine proteases. ORF2 is fused with ORF1 to express the 147 kDa replicase protein by minus one transcoding. ORF3 encodes a coat protein (CP). ORF5 encodes a coat protein read-through protein (CP-RTD) through the read-through strategy.

### 2.2. Characterization of CVEV-DT1-Derived siRNAs

Identification of the viral small interfering RNAs (vsiRNAs) provides strong evidence for the existence of this virus. Hence, the profile of vsiRNAs derived from CVEV-DT1 was characterized. A total of 8209 vsiRNAs were found to map perfectly to the genome of CVEV-DT1 with zero mismatch; among them, the 22-nt size was the most dominant class. Further analysis of the vsiRNA strand polarity revealed a slightly higher preference for the viral sense chain of CVEV-DT1, accounting for approximately 56.47% of the total vsiRNAs (Figure 1C). Additionally, a genome-wide view of vsiRNAs along the genome of CVEV-DT1 revealed the most abundant CVEV-derived siRNA matched both positive and negative strands of the ORF5 coding region located between 4983 nt and 5022 nt (Figure 1D).

### 2.3. Phylogenetic Analysis of CVEV-DT1

**Figure 2:**
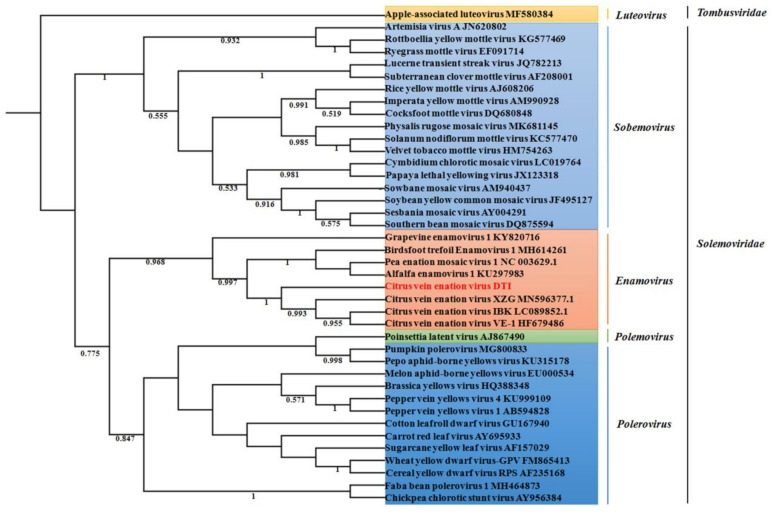
Phylogenetic analysis of CVEV and selected solemoviruses based on amino acid sequences of RNA-directed RNA polymerase using the maximum likelihood method with a bootstrap test of 1000 replicates. Apple-associated luteovirus from the *Luteovirus* genus served as the outgroup.

A phylogenetic tree, based on the multiple alignment of the amino acid sequence of the RNA-directed RNA polymerase domain derived from representative members in the *Solemoviridae* family, using the maximum likelihood algorithm, was constructed to reveal the evolutionary relationship of CVEV-DT1. The results showed that CVEV-DT1 clustered together with grapevine enamovirus 1, birdsfoot trefoil enamovirus 1, pea enation mosaic virus 1 and alfalfa enamovirus 1 in the clade of the *Enamovirus* genus, within the family *Solemoviridae*. Furthermore, CVEV-DT1 was in a subgroup together with other reported isolates of CVEV (Figure 2).

**Figure 3:**
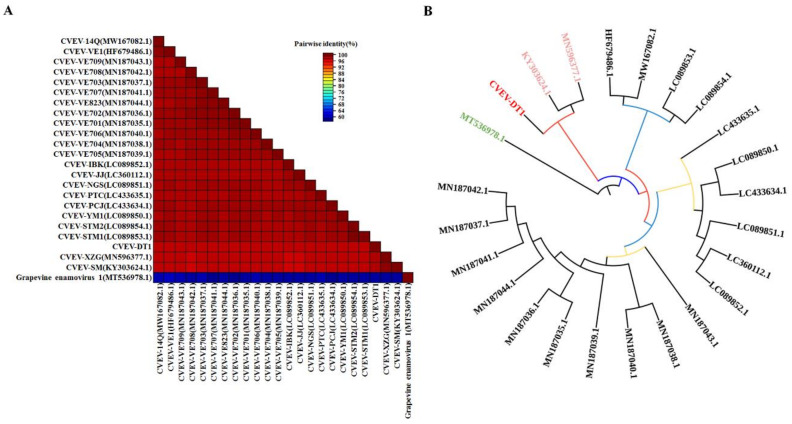
Phylogenetic analysis of all known CVEV isolates. (**A**) The SDT interface: color-coded pairwise identity matrix generated from CVEV sequences. Each colored cell represents a score of percentage identity between two sequences (one indicated horizontally to the left and the other indicated vertically at the bottom). (**B**) Phylogenetic trees derived from the CVEV whole genome nucleotide sequences using the maximum likelihood method. Grapevine enamovirus 1 served as the outgroup.

Consistently, whole genome sequence alignments of the 23 CVEV isolates collected from the NCBI database identified that all CVEV isolates, including CVEV-DT1, shared high nucleotide similarity, ranging from 96.9% to 98.9% (Figure 3A). Additionally, the five predicted ORF products had a high amino acid identity of 98.84% to 99.84% between different CVEV isolates (Table 1). The results suggest that the CVEV population is genetically very homogeneous. A phylogenetic tree was constructed for these CVEV isolates, based on full-length sequences, to further evaluate the evolutionary relationship among different CVEV isolates. The results indicated that these isolates could be divided into two subgroups (Figure 3B). Three isolates from China (CVEV-XZG, CVEV-SM and CVEV-DT1) were closely related and clustered together in a same group, suggesting a genetically variation in those isolates from other countries.

### 2.4. Population Variation Rate and Evolutionary Analysis of CVEV

**Figure 4:**
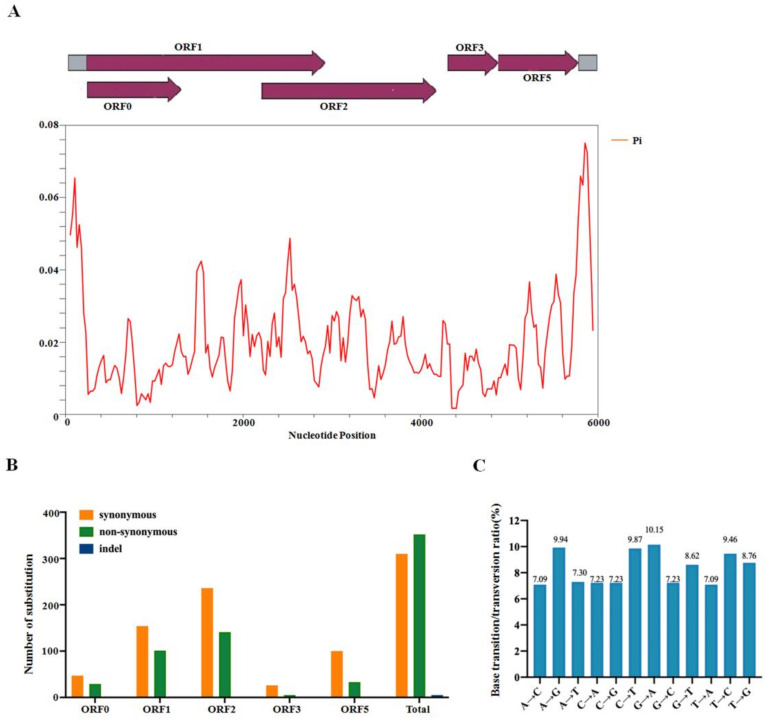
Genome-wide variation in the CVEV genome (**A**) The distribution of genetic variation evaluated along the whole genome of CVEV by nucleotide diversity (π). The CVEV genome structure was used as a reference for displaying the variation rate of each ORF region. The window length was 100 nt wide, slide by 25 nt intervals. (**B**) The substitution numbers of five ORFs (indel, non-synonymous and synonymous) in the CVEV genome were shown, respectively. (**C**) The maximum composite likelihood method was used to estimate the base transition and transversion ratio of CVEV genome.

Previous reports have identified that various mutations have occurred in the population of poleroviruses in the process of adapting to different hosts and vectors. However, a comprehensive profile of variation mapped to some species or specific proteins derived from enamoviruses is not yet available. To further accurately assess genomic variation along the whole CVEV genome, the overall nucleotide diversity index (π) of all the sites in these CVEV genomes was estimated using the DnaSP software. The results showed that the variation rate of the CVEV whole genome was less than 8%. Additionally, the average value of π was confirmed to be 0.01981. The peaks for the highest variation were located within the 5′-terminal and 3′-terminal non-coding regions, while the regions within ORF0 and ORF3 indicated relatively lower π values (Figure 4A).

Accordingly, mutations that preferentially accumulated in each CVEV ORF were analyzed. The number of non-synonymous mutations within ORF0, ORF1, ORF2, ORF3 and ORF5 were 29, 101, 187, 5 and 33, respectively, while 47, 154, 190, 26 and 100 synonymous mutations happened in each ORF (Table 1). Intriguingly, indels were not observed in all five ORFs of CVEV. These results revealed that synonymous mutations mainly occurred in ORF1, ORF2 and ORF5, and non-synonymous mutations mainly occurred in ORF1 and ORF2, suggesting ORF0 and ORF3 represent the most genetically stable genome area (Figure 4B).

Meanwhile, the maximum composite likelihood method was used to analyze the transition and transversion ratio of each site in the CVEV genome. The findings revealed that the transitions of C↔T (19.33%) and A↔G (20.09%) were higher than the transversions of A↔T (14.39%), G↔T (17.38%), A↔C (14.32%), and C↔G (14.46%) (Figure 4C), indicating biases for the transitions of C↔T and A↔G.

### 2.5. Recombination Analysis of CVEV 

The Datamonkey website was used to investigate the types of options that act on the five CVEV ORFs. The results showed that the dn/ds ratios of all five ORFs were significantly less than 1, indicating that all CVEV genes were under negative or purified selection (Table 2). To further detect putative recombination events, the whole genome sequences of the 23 CVEV isolates were scanned using RDP4 software. As a result, no recombination events were identified among the CVEV isolates that satisfied the threshold of at least four of the seven recombination detection algorithms and had a high acceptable *p*-value < 0.05.

### 2.6. Disorder across CP of CVEV-DT1

**Figure 5:**
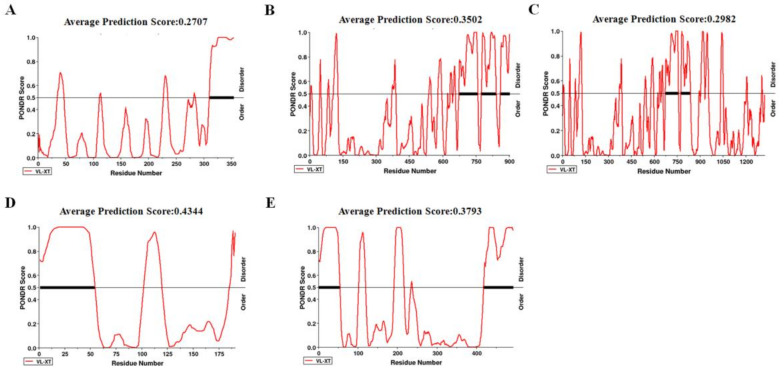
Disorder analysis of CVEV-encoded proteins using IDR online forecasting site with the algorithm VL-XT. The protein disorder of ORF0 (**A**), ORF1 (**B**), ORF2 (**C**), ORF3 (**D**) or ORF5 (**E**) was predicted, respectively.

Numerous intrinsically disordered proteins or intrinsically disordered protein regions (IDRs) can drive phase separation. To elucidate which protein was involved in phase separation, protein disorder was predicted in all five ORFs of CVEV-DT1 via the online tool PONDR, using VS-XT as the predictor (Figure 5). The results revealed that CP is a highly disordered protein that contains, overall, 45.03% disordered regions with one RDR in the 5′-terminal. Another highly disordered protein is CP-RTD, with 35.70% disordered regions.

**Figure 6:**
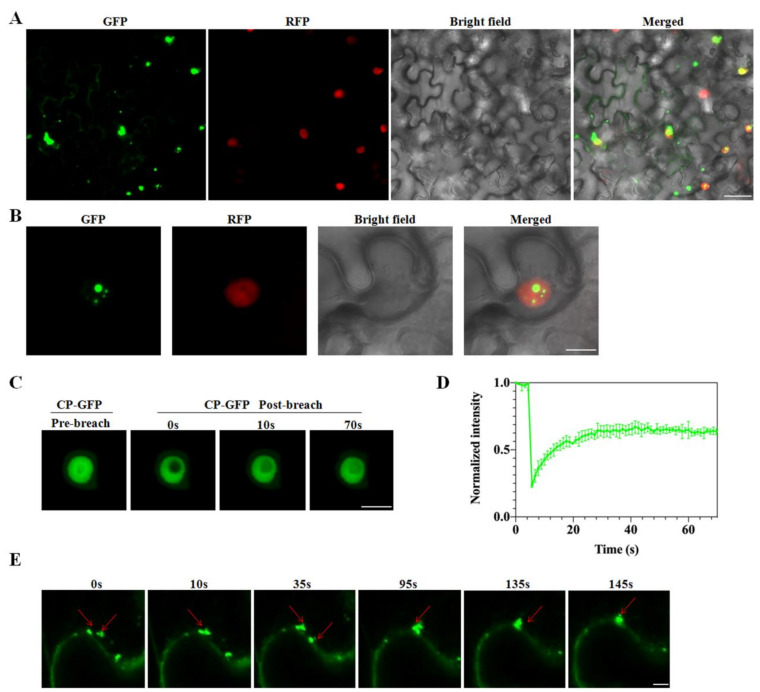
CVEV-DT1 CP protein forms liquid-like granules through liquid-liquid phase separation (LLPS) in vivo. (**A**) Subcellular localization of CVEV CP in H2B transgenic *N. benthamiana* leaves. Scale bars = 50 μm. (**B**) Subcellular localization of CVEV CP in H2B transgenic *N. benthamiana* leaves. Scale bars = 5 μm. (**C**) Representative image showing fluorescence recovery of CP-GFP granules after photobleaching (FRAP) in *N. benthamiana* leaf epidermal cells at 48 h. Scale bar = 5 μm. (**D**) FRAP recovery curves of CP-GFP granules. The intensity of each granule was normalized according to its pre-bleached fluorescence. The data were presented as mean ± SD of 3 granules. (**E**) Confocal images showing dynamic fusion of CP-GFP granules in *N. benthamiana* leaf epidermal cells. Red arrows indicate fusion of CP-GFP granules. Scale bars = 50 μm.

Based on the average prediction score, we chose CVEV-DT1 CP to test whether it could drive liquid-liquid phase separation. Firstly, the subcellular localization of CVEV-DT1 CP in *N. benthamiana* leaves was evaluated. Confocal imaging showed that 35S-CP:GFP formed spherical granules of different sizes, mainly in the cell nucleus as well as being partly distributed throughout the cytoplasm at 2 dpi (Figure 6A,B). Then, we examined whether the CVEV-DT1 CP spherical granules had liquid properties through fluorescence recovery after photobleaching. Approximately 56% of the CP-GFP granule signal gradually recovered within 70 s after photobleaching (Figure 6C,D). Further time-lapse confocal imaging indicated that the CVEV-DT1 CP granules moved rapidly and fused with each other (Figure 6E). Taken together, these results confirmed that the CVEV-DT1 CP protein could form granules through liquid-liquid phase separation in vivo.

### 2.7. CVEV-DT1 P0 as an Inducer of Hypersensitive-Response-like Cell Death

**Figure 7:**
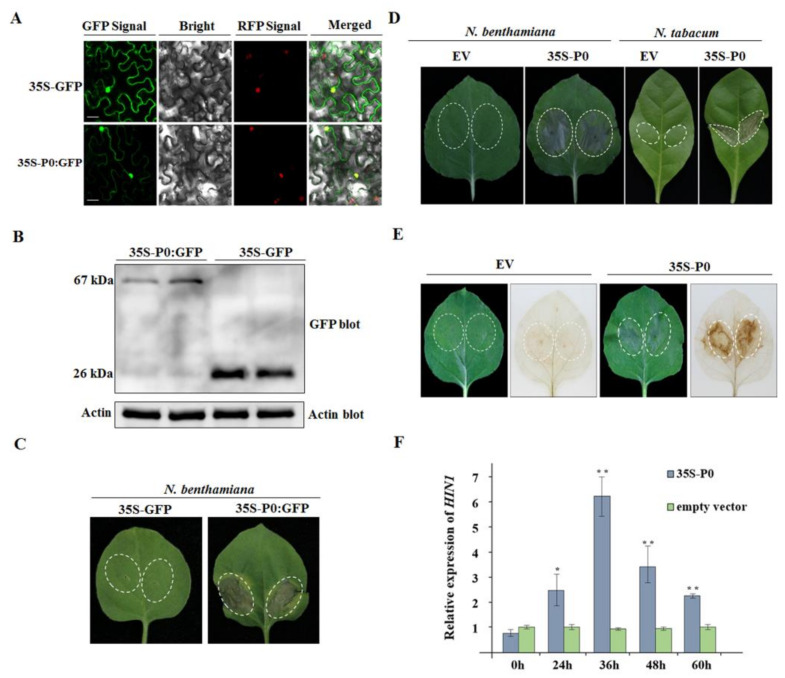
HR-like response induced by transient expression of CVEV-DT1 P0 in *N. benthamiana* leaves. (**A**) Subcellular localization of CVEV-DT1 P0 protein in H2B transgenic *N. benthamiana* inoculated with 35S-P0:GFP constructs at 36 h post-infiltration. (**B**) Detection of the expression of P0-GFP fusion protein by Western blotting. (**C**) HR-like cell death induced by P0-GFP fusion protein in *N. benthamiana*. (**D**) HR-like cell death induced by P0 protein in *N. benthamiana* and *N. tabacum* leaves inoculated with 35S-P0 constructs at 3 days post-infiltration (dpi). (**E**) Detection of H_2_O_2_ accumulation in *N. benthamiana* leaves inoculated with 35S-P0 constructs at 2 dpi by 3,3-diaminobenzidine (DAB) absorption method. (**F**) Analysis of the transcriptional level of *HIN1* in *N. benthamiana* leaves inoculated with 35S-P0 constructs by qRT-PCR. Asterisks (* or **) indicate levels of significant difference (*p* ≤ 0.05 or *p* ≤ 0.01).

In line with the phenomenon that viral pathogen invasion produces a necrosis phenotype in non-host plant species to trigger plant immunity, some evidence confirmed that viral protein can elicit hypersensitive-response-like cell death in the vicinity of the entry points. To determine whether the CVEV P0 protein is an inducer of cell death, a transient expression construct of 35S-P0:GFP under the control of the 35S promoter from cauliflower mosaic virus (CAMV) was produced and transformed into *A. tumefaciens*. Transgenic *N. benthamiana* overexpressing the RFP-tagged nuclear histone protein H2B was inoculated with 35S-P0:GFP. As is shown in Figure 7A, examination of the GFP and RFP signals by confocal microscopy at 36 h after inoculation revealed P0:GFP fusion protein located in both the nucleus and cytoplasm. Further Western blot analysis confirmed the expression of P0:GFP in inoculated leaf tissues (Figure 7B). Subsequently, a continuous observation of symptoms identified obvious necrotic spots elicited by P0:GFP in inoculated leaves at 72 h after inoculation (Figure 7C).

To avoid the influence of GFP fusion on the function of P0, a construct of 35S:P0 was generated. Three to four days after agroinfiltration into *N. benthamiana* and *N. tabacum*, the cell death phenotype were observed in the infiltrated areas (Figure 7D). In order to further determine whether the occurrence of the necrosis reaction was related to a burst of reactive oxygen species (ROS), production of H_2_O_2_ was assayed using the DAB absorption method. The results showed that only 35S:P0-infiltrated leaf tissues of *N. benthamiana* became brown at two days post-inoculation (dpi), while the leaves inoculated with an empty vector were transparent, indicating that the transient expression of P0 protein stimulated the accumulation of H_2_O_2_ (Figure 7E). Subsequently, the analysis by qRT-PCR revealed that the expression levels of the HR-related marker gene *HIN1* (the harpin-induced 1 gene) were remarkably elevated in 35S:P0-infiltrated *N. benthamiana* leaves at 24 h, 36 h, 48 h and 60 h after inoculation, compared to those in leaves inoculated with the empty vector (Figure 7F).

### 2.8. CVEV-DT1 P0 Elicits Cell Death in a PVX Expression Assay

**Figure 8:**
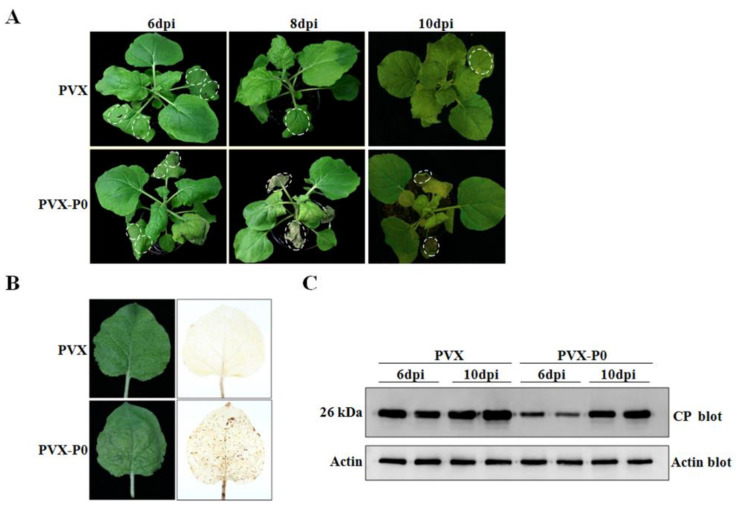
HR-like response induced by CVEV-DT1 P0 in a PVX expression assay. (**A**) Symptoms produced in *N. benthamiana* plants agroinfiltrated with PVX-based vector expressing CVEV P0 protein. (**B**) Detection of H_2_O_2_ accumulation in *N. benthamiana* leaves by DAB absorption method at 8 dpi. (**C**) Detection of the expression of PVX CP by Western blotting.

To further investigate the role of P0 protein in inducing cell death in non-host species, a recombinant PVX-based vector expressing CVEV P0 was introduced into *N. benthamiana* plants. As shown in Figure 8A, the agroinfiltrated leaves showed severe HR-like necrosis, and the scattered cell death spots had spread systemically into the newly emerging upper leaves at 8 dpi. Severe cell death and collapse of tissue was observed at 10 dpi. In contrast, *N. benthamiana* plants inoculated with the wild type PVX vector exhibited mild curly leaf symptoms in systemically infected leaves, and no evidence of necrosis was observed in the inoculated area until 10 dpi (Figure 8A). In order to further validate the necrotic phenotype induced by PVX-P0, this was correlated with H_2_O_2_ accumulation; the emerging upper leaves showing sporadic HR-like lesions were collected from PVX-P0-inoculated *N. benthamiana* plants at 8 dpi and stained using a DAB reagent. The results showed that a burst of H_2_O_2_ was accompanied with CVEV P0 expression (Figure 8B). Further Western blotting specific for PVX-CP indicated that the accumulation of PVX within the upper leaves was remarkably reduced in PVX-P0-inoculated plants at 6 dpi, compared to the control inoculated with PVX empty vector. At 10 dpi, no obvious differences in the accumulation of PVX were observed in the upper leaf tissues of *N. benthamiana* plants infected with PVX-P0 or a PVX empty vector (Figure 8C). We speculated that the HR induced by CVEV P0 might prevent systemic invasion of PVX during early infection. 

### 2.9. CVEV-DT1 P0 Is a Weak Suppressor of PTGS

**Figure 9:**
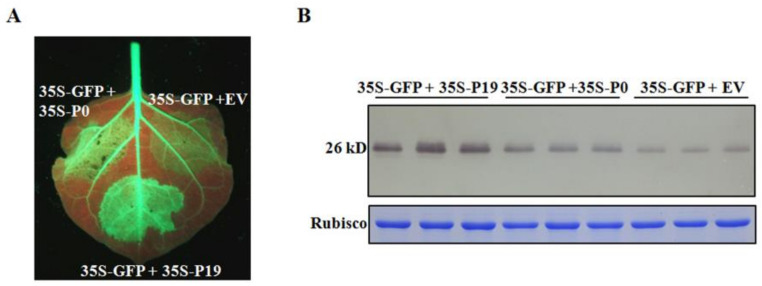
Suppression of GFP local silencing by CVEV-DT1 P0 protein. (**A**) Effect of CVEV-DT1 P0 protein on GFP local silencing in transgenic *N. benthamiana* line 16C expression GFP. The photographs were taken at 3 dpi under UV light. (**B**) Detection of the accumulation of GFP protein extracted from agroinfiltrated patches shown in (**A**).

Analysis of the amino acid sequence of the CVEV P0 protein identified a conserved F-box domain (52-LLPYVLx10P-67), suggesting that it might also have suppressor activity of RNA silencing. In order to determine whether CVEV P0 can inhibit PTGS, the agroinoculation groups of 35S:GFP + 35S:P0, 35S:GFP + 35S:P19 and 35S:GFP + empty vector, were inoculated into the leaves of the transgenic *N. benthamiana* line 16C. The *A. tumefaciens* strain expressing tomato bushy stunt virus (TBSV) p19 protein was introduced as a positive control in the assay. By 3 dpi, the intensity of GFP fluorescence in inoculated leaves was examined under UV light. Weak GFP fluorescence was observed in the infiltration areas inoculated with the negative control (35S:GFP + empty vector), indicating that partial GFP silencing had occurred. At this time, the GFP fluorescence in the 35S:GFP + 35S:P0 infiltrated region was slightly stronger than that in the negative control, but was significantly reduced compared to the GFP signals observed in the agroinoculation group of 35S:GFP + 35S:P19 (Figure 9A). Further Western blot assay specific for GFP indicated that leaf patches inoculated with the 35S:GFP + 35S:P19 combination had the highest GFP protein accumulation. Leaf patches inoculated with the 35S:GFP + 35S:P0 combination had lower GFP accumulation, but this was still significantly higher than that of the leaf patches inoculated with the 35S:GFP + empty vector combination (Figure 9B). This is consistent with the results obtained from GFP fluorescence observation. Therefore, these results strongly suggest that the CVEV P0 protein is capable of suppressing local gene silencing.

## 3. Discussion

In our study, a new full-length isolate of CVEV with 5983 nt was identified from Zhejiang province, China. Our screening for the presence of citrus pathogens revealed a mixed infection of CVEV-DT1 with CTV and CEPV in a field sample. In line with our observation, various coinfections of diverse viruses have been progressively identified in plant hosts following the application of high throughput sequencing [22,23,24]. Hence, it is highly challenging to identify the actual pathogen that produce field symptoms using a reverse genetics tool based on an infectious clone derived from the viral genome. For CVEV, Xu et al. demonstrated that *C. aurantium* plants inoculated with the infectious clone of CVEV appeared to have small auricular-typed outgrowths in the lateral veins of the leaves, fulfilling the Koch’s postulates test [25].

Citrus plants showing vein enation disease were first discovered in 1953 [6]; however, the causal agent was only identified and named as CVEV in 2013 [3]. More recently, twenty-two whole genome sequences of CVEV isolates were successively reported worldwide. Although its pathogenicity had been determined, little has been explored regarding the genome-level variability and genomic function of CVEV. Previous reports demonstrated geographic isolation which has shaped the genomic structure of the viral population [26,27,28]. Herein, phylogenetic analysis based on the whole genome identified all known CVEV isolates including CVEV-DT1 clustered into two divergent subgroups, indicating a clear geographic segregation. These isolates from China were most closely related and were separated from those isolated from other countries. Hence, it is tentatively assumed these CVEV isolates collected from China might have the same evolutionary origin. Even so, a larger database based on the CVEV full-length genome sequences from diverse world distributions would aid in further predicting the CVEV geographic origin.

RNA viruses usually exhibit a relatively high degree of within-species genetic variation to maintain functionality in adapting to a diverse range of abiotic and biotic stresses [29,30,31]. Analysis of genetic variation across the viral population can provide insight to better understand its epidemiology, evolution and host adaptation. Previously the genomic variability of a single nucleotide among cotton leafroll dwarf virus (CLRDV) isolates, that belong to the genus *Polerovirus*, was estimated to be π = 0.02831, from analysis of 17 full-length genomic sequences (*n* = 17) [32]. Our findings estimated the genomic variability for CVEV populations to be π = 0.01981 (*n* = 23), suggesting that the CVEV population is less variable than the CLRDV. Mutation and recombination are among the strongest forces driving plant virus variation [33]. In most poleroviruses, mutations preferentially accumulate in P0 and CP-RTD coding regions [2]. However, our findings showed that ORF1 and ORF2 genes encoded by CVEV were more variable compared to other encoding regions of the genome. Noticeably, recombination spots in some poleroviruses are located within the RdRp and the 5′ region of ORF1 [34,35]. Although no recombination events were detected among these CVEV isolates, hypervariable areas within ORF1 and ORF2 might have the potential to promote recombination among different viral isolates or species.

The selective force of different viral coding regions usually varies. For instance, the coding region of ORF4 of maize yellow mosaic virus (MaYMV) was under positive selection pressure, while the rest of the genes were under negative selection pressure [26]. For poleroviruses, similar report revealed that negative selection was the dominant selective force driving genomic variation in the viral coding regions of CLRDV [32]. However, analysis of the mean dn/ds ratios revealed that all coding regions of CVEV were under negative selection pressure. Our findings were consistent with those of the study described previously that indicated all genes encoded by rice stripe mosaic virus (RSMV) were controlled by negative selection pressure [28]. Some aphid-transmitted viruses such as cucumber mosaic virus (CMV) were subject to severe bottlenecks during horizontal transmission by vectors, which contributed to a dramatic loss of genetic diversity in viral populations [36]. Hence, it is reasonable to assume aphid transmission may be involved in limiting CVEV genetic variation.

Many disordered proteins have been shown to form liquid-like condensates through liquid-liquid phase separation (LLPS), which mediates diverse biological functions [37]. LLPS related to viral proteins also plays important roles in the formation of membraneless compartments to form viral replication factories and promote the assembly of viral machinery during viral infection [38,39]. For viruses, the evidence currently indicates that LLPS mainly applies to RNA viruses that infect animals [40,41]. However, increasing studies have revealed that LLPS is associated with plant viruses. Recently, Fang et al. reported that a phosphoprotein encoded by negative-sense barley yellow striate mosaic virus could form liquid-like granules through LLPS in vivo [42]. The movement protein from positive-sense pea enation mosaic virus 2, which belongs to the *umbravirus* genus in the *Tombusviridae* family, could drive LLPS with cellular factors to modulate virus-host interactions [43]. Our study demonstrates that the CP of CVEV-DT1 undergoes LLPS, suggesting it might have a flexible three-dimensional structure to interact with multiple partners in the host. In line with our findings, previously the nucleocapsid (N) protein from the positive-sense SARS-CoV-2 (severe acute respiratory syndrome coronavirus 2) has been shown to form condensates with viral genomic RNA [44,45]. Furthermore, capsid proteins from the positive-sense Dengue virus and Zika virus were demonstrated to dock to liposomes and form liquid droplets with nucleic acids [46]. Although our results has expanded the knowledge of CVEV-DT1-host interactions occurring through LLPS, further experiments will be required to elucidate whether viral genomic RNA or cellular factor(s) are involved in modulating LLPS mediated by the CVEV-DT1 CP.

Pathogen proteins can induce HR-like necrosis of tissue in plants through effector-triggered immunity or gene-to-gene recognition [47,48,49]. Previously, several P0s from ploeroviruses, including TuMV (P0^Tu^), potato leafroll virus and cucurbit aphid-borne yellow virus, were reported to serve as an inducer of HR and elicited the typical cell death phenotype in *N. glutinosa* accession TW59 [47,50]. Further evidence showed that P0^Tu^ induced an HR-like immune response through interaction with a resistance protein known as RPO1 (resistance to poleroviruses 1) [50]. Our observation indicated that *N. benthamiana* leaves inoculated with a binary vector or a recombinant PVX-based vector carrying the P0 gene of CVEV-DT1 appeared to exhibit HR-type necrosis, accompanied with a burst of H_2_O_2_ and induced expression of *HIN1*. It is possible that CVEV-DT1 P0 is recognized by an unknown R protein of *N. benthamiana*.

Previous studies have revealed that poleroviruses have employed P0 proteins as viral suppressors of PTGS to counteract the host antiviral silencing activity by targeting AGO1 [51,52]. Subsequently, Li et al. reported that the brassica yellows virus (BrYV) P0 protein could interact with SKP1 to degrade AGO1 [19]. Additionally, Michaeli et al. revealed that the P0 protein encoded by turnip yellows virus (TuYV) could modulate AGO1 degradation via the delivery of ER-bound AGO1 to the vacuole [53]. Similar to these P0s from poleroviruses, the P0 of PEMV1 was demonstrated to strongly inhibit PTGS through a conserved mode of action upon mediating AGO1 destabilization [21]. Our primary findings showed that the CVEV-DT1 P0 protein had weak suppressor activity but failed to interact with SKP1, suggesting an as-yet-unknown host factor could interact with CVEV-DT1 P0 and is essential for its silencing suppressor activity.

## 4. Materials and Methods

### 4.1. Plant Materials

The CVEV-DT1 isolate was obtained from a field citrus sample (*Citrus sinensis,* blood orange) showing scattered enations in the abaxial surface of leaves and the corresponding indentations in the adaxial surface, collected from Lishui district of Zhejiang Province, China, in June, 2014.

### 4.2. Small RNA Deep Sequencing and Data Processing

Total RNA from DT-l samples was extracted from leaf tissues using the TRIzol reagent (Invitrogen, Carlsbad, CA, USA) following the manufacturer’s guidelines. The quality of the purified RNA was evaluated using a spectrophotometer (Nanodrop, Thermo Fisher Scientific, Waltham, MA, USA). A 10 μg mixed RNA preparation was used for construction of the sRNA library and small RNA sequencing using the Hiseq2500 platform. Raw reads were filtered and cleaned with an in-house Perl script. Then, the 18–28 nt reads consisting of trimmed sRNA sequences were collected for genome assembly using the Velvet program (EMBL-EBI, Cambridge, UK), with a parameter of 17 nucleotides set as the minimal overlapping length (k-mer), to join two sRNAs into a contig. BLASTn alignments were carried out to identify contig sequences in the NCBI database.

### 4.3. RT-PCR Validation, Full-Length Genome Amplification, and Sequencing

RT-PCR assays were performed to amplify viral genomic sequences using primers based on the obtained contig sequences and the known CVEV-VE1 sequence, as described previously [3]. The 5′- and 3′-terminal sequences of the CVEV-DT1 were obtained via a RACE using SMARTer ™ RACE cDNA Amplification Kit (Clontech, Mountain View, CA, USA) according to the manufacturer’s instructions. The resulting fragments were assembled into the full-length CVEV-DT1 sequence using the DNAStar Lasergene package (Version 7.1.0, DNAStar Inc., Madison, WI, USA).

### 4.4. Phylogenetic Analysis

Phylogenetic analysis was performed based on the MUSCLE multiple sequence alignment available in the SDT V1.2 software. The phylogenetic tree was constructed via the maximum likelihood (ML) method using MEGA X software and evaluated via a bootstrap test with 1000 replicates. The sequences for the phylogenetic analysis were downloaded from the NCBI database.

### 4.5. Diversity and Variation Analysis

Nucleotide diversity was analyzed using DnaSP6, setting the window length at 100 and the step length at 25. The pi value was used to evaluate the mutation rate of a single nucleotide across the genome. The base transfer/transversion ratio for the CVEV genome and the occurrence of synonymous and non-synonymous mutations on different CVEV ORFs were analyzed using the MEGA X software.

### 4.6. Genomic Sequence Analysis

The selection of different CVEV ORFs was estimated using the Datamonkey website using three methods: the fixed effects likelihood (FEL) method using the maximum likelihood (ML) method; the fast, unconstrained Bayesian approximation (FUBAR) method using the Bayesian method; and the single likelihood ancestor counting (SLAC) method using a combination of the maximum likelihood (ML) and counting methods. The average dn/ds ratio of five CVEV ORFs was calculated based on SLAC method. Analysis of recombination sites in CVEV sequences was carried out using the Recombination Detection Program (RDP) 4 software.

### 4.7. Prediction of IDR

The intrinsically disordered regions (IDRs) were predicted with the online tool PONDR (http://www.pondr.com/ (accessed on 18 April 2022)) with default parameters. A value above 0.5 represents disorder.

### 4.8. Fluorescence Recovery after Photobleaching (FRAP) Assay

FRAP of the CP-GFP body in *N. benthamiana* leaf epidermal cells was performed using a laser scanning confocal microscope FV3000 (Olympus, Tokyo, Japan); ×40 objective. bodies were bleached using a laser intensity of 25% at 488 nm with 100 iterations. Images were acquired using FV31S-DT (Olympus, Tokyo, Japan) software.

### 4.9. Vector Construction and Plant Inoculation 

To transiently express the CVEV P0 protein, the coding sequence of CEVE P0 was amplified from the citrus sample DT1 using the RT-PCR method, and the resulting PCR fragment was sequenced to avoid base errors; it was then cloned into the empty binary vector pCHF3 or a recombinant pCHF3 vector (containing an eGFP reporter gene) that was digested using the restriction enzymes KpnI and BamHI. These recombinant pCHF3 expression constructs were introduced into the *A. tumefaciens* strain C58C1 via the electroporation method.

To express the CVEV P0 protein via the PVX-based vector, the coding sequence of CEVE P0 was inserted into the ClaI/SalI digested PVX vector PGR107. The PVX-derived expression construct was transformed into *A. tumefaciens* GV3101 using electroporation.

For the transient expression of the CVEV CP protein, the coding sequence of CEVE CP was amplified from the citrus sample DT1 using RT-PCR, and the resulting PCR fragment was sequenced to ensure that no base errors were introduced; then, it was cloned into the binary recombinant pGD vector (containing the eGFP reporter gene) that was digested with the restriction enzymes SacI and BamHI.

*A. tumefaciens* cultures harboring different constructs were cultured overnight until the OD 600 reached approximately 0.8; then, they were resuspended in the induction buffer (10 mM MES, pH 5.7, 10 mM MgCl_2_, 200 mM acetosyringone) to a final OD 600 = 1.0. The agrobacterium cultures were individually inoculated into the leaves of *N. benthamiana* plants at the four-leaf-stage using 1-mL needleless syringes. Inoculated plants were cultured in a growth chamber at 25 °C under a 16 h/8 h light/dark cycle.

### 4.10. Subcellular Localization

The transformed *A. tumefaciens* cultures were infiltrated into leaves of four-leaf-stage *N. benthamiana* plants. Fluorescence was observed and photographed by confocal microscopy (Leica TCS SP5) at 36–48 h after infiltration.

### 4.11. PTGS Assay

The agrobacterium cultures carrying the pCHF3 empty vector, 35S-GFP, 35S-P19 and 35S-P0 were prepared, and the final OD600 was adjusted to 1.0. The following groups of empty vector + 35S-GFP, 35S-P0 + 35S-GFP and 35S-P19 + 35S-GFP were mixed according to a 5:4 ratio and inoculated into the seedlings of transgenic 16c *N. benthamiana* plants carrying the GFP gene. GFP fluorescence was detected under a hand-held 100W, long-wave UV lamp (UV products, Upland, CA, USA) and photographed using a Canon EOS 70D camera.

### 4.12. H_2_O_2_ Detection in Plants

The leaves were carefully cut and put into a 50 mL tube, and the prepared 1 mg/mL DAB (3,3’-diaminobenzidine) was added to the leaves. After being left at 25 °C for 8 h, the leaves were bleached with ethyl alcohol in boiling water to remove the chlorophyll completely. The leaves were then stored in 70% ethanol and photographed on a white light plate.

### 4.13. Protein Extraction and Western Blotting 

Total protein was extracted from the leaf tissues using an SDS-urea buffer. The protein was separated by electrophoresis in 12.5% SDS-PAGE. Antibody-specific assays were carried out with rabbit anti-PVX CP polyclonal antibodies or anti-GFP monoclonal antibodies (Epitomics, Burlingame, CA, USA) according to the description by Sun et al. [54,55].

## 5. Conclusions

In this study, we reported a new isolate of CVEV (CVEV-DT1) and demonstrated it was present in mixed infections with other RNA viruses. We further characterized the CVEV population variation based on all known CVEV full-length genome sequences, indicating that all of its genes were under negative selection pressure. We confirmed that the CP encoded by CVEV-DT1 was a disordered protein, which could form spherical granules mainly in the cell nucleus, with liquid properties. Furthermore, we also identified that the CVEV P0 protein has weak PTGS suppressor activity and could elicit HR-like cell death in tobacco plants. Our results contribute to a better understanding of genomic variation across all the reported CVEV isolates and of the functions of CVEV-encoded proteins.

## Figures and Tables

**Table 1:** Sequence identities (ID) and mutations in individual proteins or genes encoded by the 23 CVEV isolates.

Genomic Region	Amino Acids (aa)			Nucleotide (nt)		Mutations	
	Length (aa)	ID (%)	InDels	Length (nt)	ID (%)	Syn	Non
ORF0(P0)	354	99.25%	None	1065	99.34%	47	29
ORF1(P1)	902	98.86%	None	2709	98.95%	154	101
ORF2(RdRP)	1323	98.84%	None	3972	98.46%	190	187
ORF3(CP)	191	99.84%	None	576	99.47%	26	5
ORF5	494	99.25%	None	1485	99.01%	100	33

**Table 2:** Analysis of selection pressure acting upon all CVEV genes.

ORF	FEL		SLAC		FUBAR		dn/ds
	PS	NS	PS	NS	PS	NS	
0	3	13	0	4	7	12	0.3770
1	5	72	1	33	12	69	0.2963
2	11	88	0	44	20	94	0.3289
3	0	14	0	2	0	12	0.0954
5	3	41	0	20	4	36	0.1760

PS, positive/diversifying selection sites; NS, negative/purifying selection sites; dn/ds, the average ratio between non-synonymous and synonymous substitutions in each pair of comparisons, where dn/ds >1 indicates that there is positive selection pressure on gene. The statistical significance level of FEL and SLAC was a *p*-value = 0.1, and that of FUBAR was a *p*-value = 0.9.

## Data Availability

The data presented in this study are available in this manuscript.

## Supplementary Materials

The following supporting information can be downloaded at: https://www.mdpi.com/article/10.3390/ijms24010412/s1. Figure S1: Validation of the existence of viruses in the field citrus sample DT-1 by RT-PCR assay using virus-specific primers.
