# Supplementary material for: Molecular Variation and Genomic Function of Citrus Vein Enation Virus
Source: Int J Mol Sci. 2022 Dec 27;24(1):412. doi: 10.3390/ijms24010412 (PMC9820537; doi:10.3390/ijms24010412)
Supplement: Supplementary file 1 [file ijms-24-00412-s001.zip › ijms-2037755-supplementary.pdf]

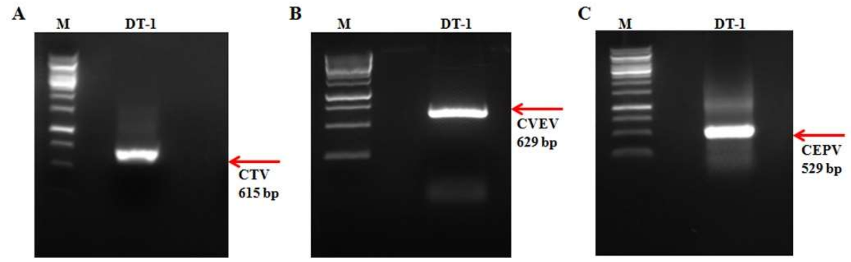

Figure S1 Validation the existence of viruses in the field citrus sample DT-1 by RT-PCR assay using virus-specific primers.
